# Active Dendrites and Local Field Potentials: Biophysical Mechanisms and Computational Explorations

**DOI:** 10.1016/j.neuroscience.2021.08.035

**Published:** 2021-09-08

**Authors:** Manisha Sinha, Rishikesh Narayanan

**Affiliations:** Cellular Neurophysiology Laboratory, Molecular Biophysics Unit, Indian Institute of Science, Bangalore, Karnataka 560012, India

**Keywords:** ion channels, degeneracy, heterogeneity, computational models, oscillations, neural plasticity

## Abstract

Neurons and glial cells are endowed with membranes that express a rich repertoire of ion channels, transporters, and receptors. The constant flux of ions across the neuronal and glial membranes results in voltage fluctuations that can be recorded from the extracellular matrix. The high frequency components of this voltage signal contain information about the spiking activity, reflecting the output from the neurons surrounding the recording location. The low frequency components of the signal, referred to as the local field potential (LFP), have been traditionally thought to provide information about the synaptic inputs that impinge on the large dendritic trees of various neurons. In this review, we discuss recent computational and experimental studies pointing to a critical role of several active dendritic mechanisms that can influence the genesis and the location-dependent spectro-temporal dynamics of LFPs, spanning different brain regions. We strongly emphasize the need to account for the several fast and slow dendritic events and associated active mechanisms — including gradients in their expression profiles, inter- and intra-cellular spatio-temporal interactions spanning neurons and glia, heterogeneities and degeneracy across scales, neuromodulatory influences, and activitydependent plasticity — towards gaining important insights about the origins of LFP under different behavioral states in health and disease. We provide simple but essential guidelines on how to model LFPs taking into account these dendritic mechanisms, with detailed methodology on how to account for various heterogeneities and electro-physiological properties of neurons and synapses while studying LFPs.

## Introduction From Alien Gadgets to Intrinsic Mechanisms

In the quest to dissect the functions of the intricate evolutionary marvel that the human brain is, researchers and engineers have been devising fascinating tools. *In vivo* neuroscience research on the role of neural activity in mediating behavior has benefitted tremendously from the works of pioneers like Richard Caton — who first performed electroencephalography and recorded electrical activity (electroencephalogram or EEG) from exposed brains of cats, rabbits and monkeys using mirror galvanometers (Caton, 1875, 1877), Adolf Beck — who observed spontaneous activity and rhythmic fluctuations that ceased after sensory stimulation (Coenen et al., 2014), Vladimir Vladimirovich Pravdich-Neminsky — who first reported evoked potentials in dogs (Pravdich-Neminsky, 1912), and Hans Berger — who improved the sensitivity of the apparatus and reported oscillations in EEG signals at frequencies around 10–25 Hz (Berger, 1929; Gloor, 1969b, a; Collura, 1993). We have come a long way from the days of EEG recordings from meditating monks (Benson et al., 1990) to the present where neurosurgeons can implant minimally invasive micro-electrocorticogram (ECoG) grids in patients to record local field potentials (LFPs) from areas as delicate as the Wernicke’s (Kellis et al., 2010). We can now record even single cell spikes from the cortical surface using flexible and organic transistors, and from deep brain areas using high-density silicon probes (Buzsaki et al., 2015; Khodagholy et al., 2016; Tybrandt et al., 2018). Thanks to such advancements in closed-loop translational neuro-electronics and biooptics (Khodagholy et al., 2013; Buzsaki et al., 2015; Chang, 2015; Krook-Magnuson et al., 2015; Rivnay et al., 2017; Cea et al., 2020; Jastrzebska-Perfect et al., 2020), what seemed like “alien gadgets” and yesterday’s science fiction are today’s reality and there has been a burgeoning effort to understand the genesis and function of extracellular spikes and LFPs, in both healthy brains and neurological disorders.

**Fig. 1:**
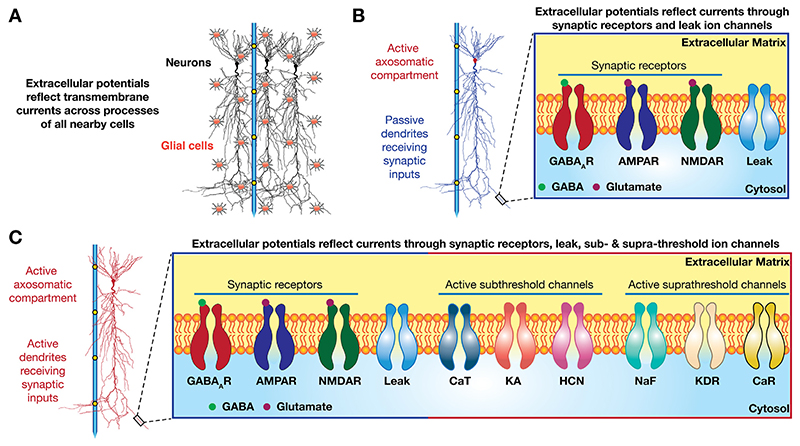
Sources of LFP. **(A)** A schematic of LFP recording from a population of principal neurons and astrocytes using a single electrode with multiple contact points that serve as recording sites. **(B)** A single recording electrode with multiple sites and a single pyramidal neuron passive dendrites and voltage-gated ion channels only in the axo-somatic compartments. A small dendritic segment is expanded to highlight various synaptic receptors and passive leak channels that contribute to the transmembrane currents. These currents are in addition to the capacitive current consequent to the two ion-conducting media (the cytosol and the cerebrospinal fluid) separated by a dielectric lipid bilayer. **(C)** Same as B but with active dendrites. An expanded view of a small dendritic segment highlights the diversity of sub- and supra-threshold ion channels/conductances that also contribute to the transmembrane currents. The morphological reconstructions are modified from neuron n123 in the open-source neuronal morphology database Neuromorpho.org (Ascoli et al., 2007).

Such an effort has progressed in parallel alongside tremendous strides taken in the field of cellular physiology of neurons and glia, both of which are now recognized to contribute to LFPs (Buzsaki et al., 2012) (Fig. 1A). Classically, LFPs were believed to reflect the synaptic inputs that were received by passive dendritic processes, with the soma as the central processing unit of a neuron (Fig. 1B). While synaptic inputs do play a crucial role in shaping LFPs, it is now well established that dendrites are certainly not mere passive recipients of inputs but express a rich repertoire of active conductances (Magee, 2000; Johnston and Narayanan, 2008; Sjostrom et al., 2008; Spruston, 2008; Narayanan and Johnston, 2012; Nusser, 2012; Major et al., 2013; Stuart and Spruston, 2015) that are equally involved in the processing of inputs and in the genesis and regulation of LFPs (Reimann et al., 2013; Sinha and Narayanan, 2015; Ness et al., 2016, 2018) (Fig. 1C).

In this review, we discuss some recent developments in this space at the circuit, cellular, and molecular scales to highlight the complexity involved in the emergence of this gestalt through the presence of active dendrites. We emphasize the need to account for active dendritic properties, gradients in the constitutive components of active dendritic structures, the dynamic nature of these components involving neuromodulation and plasticity, and ubiquitous heterogeneities that span all biological systems in analyzing LFPs. We elucidate the impact of active dendritic structures on LFPs through illustrative examples, both under physiological and pathological conditions. We provide an overview of the different methods currently being employed to address this complexity at various scales and put forward some future directions for making synergistic advances using computational and experimental approaches.

## Forms of Local Field Potentials

The constant flux of ions across the neuronal and glial cell membranes through a diverse set of ion channels, pumps, and receptors gives rise to tiny currents and voltage fluctuations which can be recorded from the extracellular matrix. This extracellularly measured voltage signal from the brain has been used to study distinct features of its ongoing activity. The high frequency content of this signal contains information about the spiking activity, reflecting the output from the neurons surrounding the recording location. The low frequency content (<∼300–500 Hz) of the voltage signal, referred to as the LFP, has been traditionally thought to provide information about the synaptic inputs that impinge on the neurons in the form of oscillations and non-rhythmic sensory-evoked events over which the spikes ride (Buzsaki et al., 2012). In this section, we high-light some of the most prominent forms of LFPs observed in the brain.

### Unitary LFPs

Unitary LFPs (uLFPs) are the field potentials generated from either a single axon or from the spiking activity of a *single unit* or presynaptic neuron, typically with a large number of its axon collaterals impinging on a small postsynaptic region. The amplitude, duration, and polarity of uLFPs can vary significantly depending on the type of presynaptic neuron, the location of its axons on the postsynatic target and anatomical connectivity through postsynaptic receptors (Swadlow et al., 2002; Bereshpolova et al., 2006; Stoelzel et al., 2008; Glickfeld et al., 2009; Bazelot et al., 2010). Computational modeling has been utilized to study the possible mechanisms underlying the uLFP signatures generated by fast-spiking inhibitory *vs* regular-spiking excitatory presynaptic single neurons (Hagen et al., 2017; Telenczuk et al., 2020b). These studies employed anatomically-constrained virtual slices/neuronal columns comprising morphologically-realistic post-synaptic neurons with experimentally determined synapse localization (Hagen et al., 2017), and trimmed axonal arborization of unitary presynaptic neuron to match the realistic size of an *in vitro* slice (Telenczuk et al., 2020b). They have provided an explanation for how a disynaptic excitatory uLFP can sometimes look like an inhibitory uLFP (Bazelot et al., 2010), highlighting the latter’s dominance when the recording electrode is located inside a predominantly inhibitory population (Telenczuk et al., 2020b). These studies also explain why it can often be difficult to separate excitatory and inhibitory uLFPs in interconnected circuits and how spike-triggered averaged LFP signatures can provide a localized measure of monosynaptic activation (Hagen et al., 2017; Telenczuk et al., 2020b). Thus, it must be emphasized that there is a critical role of axonal morphology, somato-dendritic synaptic distribution profile, anatomical connectivity, timing of input arrival, and electrode location in determining the amplitude, duration, and polarity of uLFPs in particular, and LFPs in general (Sinha and Narayanan, 2020).

### LFP oscillations

One of the most prominent set of analyses of LFPs pertains to that of its various rhythms, oscillations in different frequency bands, that have been observed in almost all regions of the brain and are associated with distinct behaviors. Brain rhythms are considered to be one of the key mechanisms for inter-regional communication, stimulus processing, and memory formation (Eckhorn et al., 1988; Nicolelis and Shuler, 2001; Buzsaki and Draguhn, 2004; Buzsaki, 2006; Kay et al., 2009), and are altered in pathological conditions (Sun et al., 2011; Gandal et al., 2012; Yordanova et al., 2013; Mably and Colgin, 2018; Yin et al., 2021). It has been argued that these neural oscillations offer distinct ways of predicting ‘what’ is going to happen ‘when’ in the sensory environment (Kay et al., 2009; Arnal and Giraud, 2012). In what follows, we briefly touch upon some of the most prominent and widely studied LFP oscillations.

#### Slow Waves: Theta and Alpha Oscillations

Theta oscillations are large amplitude oscillations observed in the ∼4–10 Hz frequency band of the LFPs in several limbic regions of the brain including the hippocampus and entorhinal cortex during awake active exploration, spatial navigation, and rapid eye movement (REM) sleep (Buzsaki et al., 1983; Leung, 1984; Buzsaki, 2002; Buzsaki and Draguhn, 2004; Colgin, 2013, 2016). While they have been well documented in rodent models, thanks to the development of virtual reality and depth stereoelectrode recording tools, theta oscillations have now also been recorded and studied in human and non-human primates (Ekstrom et al., 2005; Watrous et al., 2011; Jutras et al., 2013; Watrous et al., 2013; Bohbot et al., 2017; Goyal et al., 2020) and have been reported to code spatial distance in the absence of sensory cues (Vass et al., 2016). A recent study in humans has observations 2 distinct classes of theta oscillations (Goyal et al., 2020): low-frequency theta oscillations (∼3 Hz) that are prevalent in the anterior hippocampus with their frequency being invariant to movement speed, and high-frequency theta oscillations (∼8 Hz) that are prevalent in the posterior hippocampus with their precise frequency being correlated with the speed of movement. Thus, these two distinct oscillations may reflect nonspatial cognitive and spatial processes, respectively. A similar functional distinction of theta oscillations with reference to spatial vs. nonspatial information has also been observed in rodents along the septo-temporal axis and dorso-ventral hippocampus (Royer et al., 2010; Patel et al., 2012). Theta rhythms have been linked with path integration, spatial navigation, spatial/episodic memory formation, and memory consolidation through temporal sequences of cell assemblies of place and grid cells with cells that are a part of one assembly showing a common preference for a specific phase of these oscillations (O’Keefe and Dostrovsky, 1971; O’Keefe and Conway, 1978; O’Keefe and Nadel, 1978; O’Keefe and Recce, 1993; O’Keefe et al., 1998; O’Keefe and Burgess, 1999; Buzsaki, 2002; Harris et al., 2003; Buzsaki, 2005; Harris, 2005; Hasselmo, 2005; O’Keefe and Burgess, 2005; Huxter et al., 2008; Buzsaki and Moser, 2013; Hasselmo and Stern, 2014; Lisman et al., 2017). Loss or disruption of these rhythms has been reported to result in deficits in spatial memory and its consolidation during REM sleep (Winson, 1978; Boyce et al., 2016).

A lot of heterogeneity has been observed in theta oscillation rhythmogenesis. One form of theta oscillations originates due to the interactions between the interneurons and excitatory neurons in the medial septum – diagonal band of Broca and supramammillary nucleus (Macadar et al., 1970; Roig et al., 1970; Buzsaki, 2002) with the lateral septum playing a regulatory role (Chee et al., 2015). These oscillations travel and propagate along the septotemporal axis of the hippocampus (Lubenov and Siapas, 2009; Patel et al., 2012) and septal lesions disrupt theta oscillations in the hippocampus and entorhinal cortex (Green and Arduini, 1954; Mitchell et al., 1982). However, there is evidence that theta oscillations could also be generated locally within the hippocampal CA1 via afferents from CA3 and the entorhinal cortex, or even independent of these inputs (*in vitro*) (Buzsaki, 2002; Goutagny et al., 2009; Colgin, 2013, 2016). In the CA1, dendritic Ca^2+^ spikes have been shown to be associated with the generation of theta oscillations (Kamondi et al., 1998b). Apart from the heterogeneity in theta rhythmogenesis mechanisms, there is also heterogeneity in the modulation of spike timings through theta oscillations. For instance, pyramidal neurons and heterogeneous groups of interneurons found within the CA1 show distinct theta-modulation and phase preference (Csicsvari et al., 1999; Klausberger and Somogyi, 2008; Somogyi et al., 2014). A recent study (Navas-Olive et al., 2020) has cleverly combined computational and experimental approaches to identity the cell-type-specific intrinsic and synaptic mechanisms, as well as cell morphology, in determining theta-phase preference.

The alpha-band of oscillations (8–14 Hz) overlaps with the theta-band and is observed in the striatum, visual, somatosensory, and pre-motor/motor areas in health and disease (Haegens et al., 2011; Oswal et al., 2013; Halgren et al., 2019; Singh and Papa, 2020). It has been shown that alpha oscillations emerge in humans on closing of the eyes, and hence were thought to be associated with “cortical idling” (Berger, 1929; Pfurtscheller et al.,1996). Alpha waves thus offered new areas for the study of (un)consciousness in physiology and pathology such as locked-in syndrome (Palva and Palva, 2007; Rosburg, 2019). It was found that a progressive drug-induced loss of consciousness is tightly linked to the emergence of a hypersynchronous activity in the alpha band that was widely distributed in the frontal cortex (Supp et al., 2011). Based on the results from the same study involving stimulus-related responses to median nerve stimulation, it was suggested that blocking of intracortical communication by hypersynchronous ongoing activity could be a key mechanism for the loss of consciousness.

In contrast, LFP and spiking-activity recordings in the sensorimotor cortex show that the pulsed inhibition from these oscillations exerts a strong influence on both spike timing and firing rate. In addition, a reduction in alpha power is correlated with better discrimination performance, indicating that alpha oscillations could actively suppress irrelevant or interfering stimuli processing (Haegens et al., 2011). Also, these oscillations increase with increase in workload during retention in a short-term memory task (Jensen et al., 2002). This suggests that the alpha generating system is directly or indirectly linked to the circuits responsible for working memory. Furthermore, while the thalamocortical circuits have typically been associated with the generation of alpha oscillations in the past, studies employing LFP and spiking-activity recordings have identified intracortical alpha current generators and their potential for alpha pacemaking in the primary visual (V1), auditory (A1), somatosensory (S1), and sensorimotor cortices (Bollimunta et al., 2008; Haegens et al., 2011; Haegens et al., 2015). Thus, apart from the role of alpha oscillations in fundamental functions of attention such as irrelevant/interfering stimulus suppression, selection of salient stimuli, and in predicting forthcoming visual stimulus (van Dijk et al., 2008; Romei et al., 2010; Haegens et al., 2011; Jensen et al., 2012; Klimesch, 2012), the presence of alpha-generators across cortical depth in the neocortex suggests the involvement of these rhythms in feedforward as well as feedback processes, and is consistent with the view that alpha rhythms may be involved in parsing sensory input streams in a way that facilitates communication across cortical areas (Bollimunta et al., 2008; Haegens et al., 2015).

#### Fast Waves: Beta and Gamma Oscillations

Beta oscillations (∼13–30 Hz) in the motor cortex have been typically believed to be involved in maintaining the “status quo” or a sensorimotor state of akinesis (Engel and Fries, 2010; Khanna and Carmena, 2017). However, observations from studies involving working-memory tasks with cues and delays have led to the suggestion that beta oscillations in the prefrontal cortex (PFC) and motor cortex could potentially have 2 functions: (1) protection from interference from irrelevant stimuli during delay periods and (2) “clear out” or a stopping process to indicate the end of the cue presentation/task as beta power increases during delays and the end of a trial when working memory information needs to be erased (Swann et al., 2009; Lundqvist et al., 2011; Lundqvist et al., 2016; Lundqvist et al., 2018; Schmidt et al., 2019). Gamma oscillations (∼30–150 Hz) together with beta oscillations have also been implicated to have a role in working memory (Lundqvist et al., 2011; Lundqvist et al., 2016; Lundqvist et al., 2018). A subset of the beta band, the beta2 frequency (20–30 Hz) oscillations have been observed in the somatosensory and motor cortices during motor preparation and have been shown to depend on gap junctional coupling, with the oscillation period being determined by the *M*-type potassium current (Roopun et al., 2006). Computational analysis has helped reveal the neural mechanisms involved in transient neocortical beta oscillations in several species (Sherman et al., 2016). Within the visual cortical areas, it has been suggested that feedforward and feedback signaling use distinct frequency channels involving the slow (theta and alpha) and the fast (beta and gamma) oscillations through task-dependent dynamic (de)synchronization, and they potentially subserve differential communication requirements (van Kerkoerle et al., 2014; Bastos et al., 2015).

In the olfactory bulb (OB), odors evoke beta-gamma oscillations in the LFP (Kay et al., 2009; Martin and Ravel, 2014; Kay, 2015). These oscillations are generated through intrinsic membrane properties and the dendrodendritic interactions between glutamatergic mitral cells and local inhibitory GABAergic granule cells (GCs) (Debarbieux et al., 2003; Kay et al., 2009; Rojas-Libano et al., 2014; Kay, 2015). Activation of dendrodendritic synapses and oscillatory synchronization of cell assemblies is involved in odor memory and odor discrimination (Stopfer et al., 1997; Nusser et al., 2001; Kay et al., 2009; Lepousez and Lledo, 2013). Through biophysical modeling, it was shown that beta oscillations are produced in the OB primarily through voltage-dependent calcium channel-mediated gamma-aminobutyric acid (GABA) release, independent of N-methyl D-aspartate (NMDA) receptors, and a switch between gamma and beta oscillations can be triggered by an increase in the excitability state of a subpopulation of GCs (Osinski and Kay, 2016). These model predictions were then experimentally verified, and it was shown that even though beta oscillations rely on the same synapse as gamma oscillations, unlike gamma, they could indeed persist in the absence of NMDA receptor activation (Osinski et al., 2018). Together, (Osinski and Kay, 2016) and (Osinski et al., 2018) constitute a good example for how computational and experimental analyses could provide complementary insights about LFP genesis.

Gamma oscillations were initially debated to be not distinguishable from broadband noise (Burns et al., 2011) and are contaminated by spikes (Schomburg et al., 2012; Anastassiou et al., 2015; Ray and Maunsell, 2015), but have emerged to be involved in modulating attention, memory, temporal synchronization of inputs, and interregional communication between brain regions via cross-frequency coupling with theta-oscillations (Bragin et al., 1995; Buzsaki, 1996; Jensen et al., 2007; Jokisch and Jensen, 2007; Benchenane et al., 2011; Schomburg et al., 2014; Buzsaki and Schomburg, 2015b, a; Colgin, 2015b; Sridharan and Knudsen, 2015; Fernandez-Ruiz et al., 2021). In the visual cortex, gamma oscillations are known to be stimulus-evoked and are dependent on stimulus attributes including hue and contrast of the stimulus (Ray and Maunsell, 2011a; Shirhatti and Ray, 2018). By performing simultaneous LFP and single unit recordings from all the layers of primary visual cortex (V1), a recent study showed that the coherence of gamma oscillations and spike-LFP coupling could identify six physiological layers and further sublayers within the V1 thus bridging the mesoscopic LFPs and single unit interactions with the laminar structure of V1 (Senzai et al., 2019). There is ample computational and experimental evidence for the involvement of interneurons, especially the fast-spiking parvalbumin-expressing interneurons, in gamma rhythmogenesis (Traub et al., 1996; Wang and Buzsaki, 1996; Fricker and Miles, 2001; Bartos et al., 2007; Buzsaki and Wang, 2012). Furthermore, slow (∼30– 50 Hz) and fast (∼100–150 Hz) gamma oscillations are differentially implicated in routing flow of information, spatial temporal sequencing, novel-object place pairings, and have been hypothesized to be involved in sending segregated neuronal messages allowing a target “reader” area to disambiguate convergent inputs (Colgin et al., 2009; Colgin and Moser, 2010; Colgin, 2012; Bieri et al., 2014; Colgin, 2015a; Zheng et al., 2015; Zheng et al., 2016b; Zheng et al., 2016a; Fernandez-Ruiz et al., 2017; Trimper et al., 2017; Fernandez-Ruiz et al., 2021).

Disorganized entrainment of local circuits, with sparser dendritic arborization and lower spine density as a cellular substrate for developmental miswiring, is associated with changes in beta-gamma oscillations and neurological and psychiatric disorders (Chini et al., 2020). Increased beta-band oscillatory activity in the basal ganglia network due to resonance of intrinsic oscillations with cortical beta oscillations, with a role for the pallido-striatal feedback loop in amplifying beta oscillations, is associated with Parkinsonian motor symptoms (Koelman and Lowery, 2019; Yin et al., 2021). Deep brain stimulation has been useful in reducing these beta oscillations and improving motor function in patients with Parkin-son’s disease (Kuhn et al., 2006; Kuhn et al., 2008). Gamma oscillations are aberrant/disrupted in disorders like schizophrenia (Sun et al., 2011; Gandal et al., 2012), Alzheimer’s disease and Fragile X syndrome (Mably and Colgin, 2018). Non-invasive transcranial electrical stimulation techniques involving alternating current stimulation may turn out to be useful for resolving such disorders involving rhythmopathies (Beliaeva et al., 2021; Frohlich and Riddle, 2021; Huang et al., 2021; Riddle et al., 2021; Riddle and Frohlich, 2021).

#### Sharp Wave Ripple Complexes, Delta Waves and Spindles

During quiet wakefulness, slow wave sleep (NREM sleep) and consummatory behaviors, a complex pattern is observed in the LFPs in the CA1, CA2, and CA3 regions of the hippocampus. During these states, afferents on the dendrites of pyramidal neurons lead to their strong depolarization (Kamondi et al., 1998b; Buzsaki, 2015). This leads to the generation of a large sink (i.e., a large negative deflection in the LFP) in the *stratum radiatum* known as a sharp wave. While sharp waves are slow, they are accompanied by fast oscillations (∼100–200 Hz) in the *stratum pyramidale* known as ripples. This sharp wave ripple (SPW-R) complex lasts for ∼50–150 ms and is highly irregular (repeating with an average frequency of ∼0.5–3 Hz) (Buzsaki, 1986, 2015). Neuronal spiking and sub-threshold activity during SPW-Rs are controlled by a competition between excitation and inhibition in a sublayer-specific manner (English et al., 2014; Valero et al., 2015; Valero et al., 2017; Valero and de la Prida, 2018). During SPW-Rs, spatial memory associated theta sequences, and emotional/social memory correlates have been observed to be reactivated in a time-compressed manner (Nadasdy et al., 1999; Louie and Wilson, 2001; Cei et al., 2014; Girardeau et al., 2017; Oliva et al., 2020). There is evidence that these ripple-associated replays could be a mechanism for memory consolidation (Girardeau et al., 2009; Carr et al., 2011; Girardeau and Zugaro, 2011; Buzsaki, 2015; Fernandez-Ruiz et al., 2019; Cox et al., 2020; Oliva et al., 2020).

During NREM/slow wave sleep, cortical UP-DOWN states of alternating depolarization and hyperpolarization are observed that are associated with the generation of slow delta waves (∼1–4 Hz) and faster spindles (∼11– 16 Hz) (Steriade and Timofeev, 2003; Fuentealba et al., 2004). Sleep spindles are generated by the interaction between the GABAergic neurons of the thalamic reticular nucleus and the thalamocortical nuclei, and are coupled to the cortical delta waves (Steriade, 1993c, b, a; Steriade et al., 1993c; Steriade et al., 1993b, a; Fernandez and Luthi, 2020). There is emerging evidence for a hippocampal-neocortical dialogue during NREM sleep through SPW-Rs, delta waves, and spindles, and for their role in learning, memory consolidation and interregional transfer of information (Buzsaki, 1996; Buzsaki and Peyrache, 2013; Buzsaki, 2015; Maingret et al., 2016; Seibt et al., 2016; Antony et al., 2019; Kim et al., 2019; Todorova and Zugaro, 2019; Karimi Abadchi et al., 2020; Peyrache and Seibt, 2020; Dickey et al., 2021).

#### Epileptiform Activity: Interictal Spikes and Fast Ripples

Epilepsy is a complex set of syndromes with common behavioral correlates like recurrent seizures and convulsions associated with hypersynchronous activity in the epileptic tissue (Wyler et al., 1982; Babb et al., 1987; Cohen et al., 2002; Crunelli and Leresche, 2002; Scharfman, 2007). Different waveforms observed in the EEG in the ictal (during a seizure), interictal (between seizures) and postictal (after a seizure) activity enable clinicians to diagnose the focus and timing of seizure-stages in epileptic patients (Fisher et al., 2014). In the hippocampus and temporal cortex of epileptic humans and rodents, interictal spikes lasting 30–500 ms followed by transient high-frequency oscillations called fast ripples (250–500 Hz) are observed in the epileptogenic regions and are associated with a multitude of mechanisms depending on the form of epilepsy and the affected brain region (Dichter and Spencer, 1969a, b; Traub and Wong, 1983; Cohen et al., 2002; Bragin et al., 2004; Bernard, 2005; Urrestarazu et al., 2006; Ibarz et al., 2010; Kohling and Staley, 2011; Jefferys et al., 2012; Avoli, 2014; Valero et al., 2017; Levesque and Avoli, 2019; Levesque et al., 2019). Some of these prominent known mechanisms and associated plasticity involve a disruption of the excitation/inhibition balance, synchronous bursting of pyramidal neurons and interneurons, changes in dendritic GABAergic signaling, and dendritic channelopathies (Chen et al., 2001; Sanabria et al., 2001; Cohen et al., 2002; Su et al., 2002; Mulley et al., 2003; Whittington and Traub, 2003; Bernard et al., 2004; Shah et al., 2004; Cossart et al., 2005; Jung et al., 2007; Yaari et al., 2007; Ibarz et al., 2010; Valero et al., 2017). Such combined studies have enabled the development of several anti-epileptic drugs (Rogawski and Loscher, 2004). Computational simulations have also been used as tools to aid experiments in tackling the complex problem of epileptogenesis (Lytton et al., 2005; Traub et al., 2005; Lytton, 2008).

## Active Dendritic Conductances, Dendritic Morphology, and Lfps

With the emergence of new techniques for circuit manipulations and large scale LFP recordings, the focus of neuroscience research has shifted towards teasing apart how brain circuits work together to give rise to behavior. Introduction of ‘alien’ ion channels into neurons, such as channelrhodopsin (Boyden et al., 2005), to control the ionic transmembrane currents towards manipulating large populations of neurons synchronously has become commonplace. At the molecular level, this powerful tool is driven by an ensemble current emergent from tiny single-channel ionic currents (single channel conductance is merely ∼ 1 pS (Lin et al., 2009)), and can introduce massive perturbations to even large nervous systems manifesting as large changes in spikes and LFPs (Kim et al., 2017; Oliva et al., 2018; Fernandez-Ruiz et al., 2019; Jun and Cardin, 2020; McKenzie et al., 2021). The power of such ion channels in altering network physiology begs attention to the study of endogenous ion channels that express on neuronal and glial (Verkhratsky and Steinhauser, 2000) membranes in shaping LFPs, apart from their established roles in altering neural activity and behavior.

As the predominant recipients of afferent synaptic information, dendrites are uniquely placed to process afferent information and critically regulate spike generation in a neuron. Although dendrites were historically considered to be passive structures that were *merely* housing afferent synapses, it is now abundantly clear that dendritic structures express several ion channels, earning them the moniker *active dendrites* (Johnston et al., 1996; Poirazi and Mel, 2001; Johnston et al., 2003; Johnston and Narayanan, 2008; Major et al., 2013; Stuart and Spruston, 2015). The presence of these active components has endowed dendrites with specialized processing and propagation capabilities that manifest through strong spatio-temporal interactions across the dendritic arbor. If dendrites were not merely housing synapses and carry transmembrane proteins other than synaptic receptors, shouldn’t field potentials, composite signals that reflect transmembrane currents, be shaped by the presence of these active conductances? Here, we explore this question with reference to the different conductances that express in active dendritic structures.

### Suprathreshold dendritic conductances and LFPs

Action potentials or spikes are commonly used as a readout of neuronal responses to stimuli and of information transfer. To understand the role of neurons in generating behavioral outputs and to dissect their function in a neuronal circuit, nowadays, extracellular spike recordings using high-density electrodes and silicon probes are routinely performed from freely moving and head-restrained laboratory animals under awake, asleep, and anesthetized conditions. Most extracellular spikes are of a very short duration (typically < 1–2 ms, >500 Hz) and therefore are filtered out from the LFP (typically < 350 Hz). Yet, a computational study has highlighted that spike-related currents can impact the LFP even below 50 Hz (Reimann et al., 2013). Further computational and experimental studies have shown that a component of the spikes “bleeds through” or ‘contaminates’ the LFP in the lower frequency harmonics of the population spike frequency, especially in the epsilon band (90–150 Hz) and thus a stimulus-triggered spike signature can also be obtained in the LFP (Ray et al., 2008; Reichinnek et al., 2010; Ray and Maunsell, 2011b; Belluscio et al., 2012; Scheffer-Teixeira et al., 2013; Schomburg et al., 2014; Anastassiou and Koch, 2015; Taxidis et al., 2015; Kuokkanen et al., 2018). This is especially useful when multiple electrodes are not available to sort the spikes into individual units and if the focus of the study is to perform a population-level analysis of neuronal activity. However, such population-level analysis can lead to inaccurate conclusions in case the spikes are from a heterogeneous network of neurons, so one must be careful while making assumptions about the homogeneity of the underlying network of neurons (Valero et al., 2015; Valero and de la Prida, 2018). Further, the average of stimulus-triggered LFPs (STA-LFPs), has been used to show that units across several regions can become transiently synchronized specifically during LFP oscillations, even if their spikes are uncorrelated during non-oscillatory periods (Murthy and Fetz, 1996). Using intracellular recordings from the cortex of awake rats, STA-LFP has also been shown to represent the synchrony between the mean synaptic activity of the population and the membrane potential of the single neuron (Okun et al., 2010). But the STA-LFP must be used with caution as highlighted by a detailed analysis of the shape of the STA-LFP from the monkey visual cortex (Ray and Maunsell, 2011b). This study showed that network rhythms can influence the relationship between STA-LFP and functional connectivity, giving a false impression of a traveling LFP wave.

Extracellular spikes reflect the intracellular action potentials that are initiated above a certain membrane voltage threshold. They are a result of a complex interplay between inputs from synaptic receptors, intracellular calcium signaling, and various voltage-gated ion channels. Most *in vivo* studies focus on spikes recorded from the peri-somatic regions that express several suprathreshold-active spiking conductances such as voltage-gated Na^+^, K^+^, and Ca^2+^ channels. But it is now well established that neuronal dendrites are also endowed with a plethora of suprathreshold active conductances that can both initiate spikes and lead to their active (back)propagation in the dendrites (Stuart and Sakmann, 1994; Johnston et al., 1996; Hoffman et al., 1997; Golding and Spruston, 1998; Schiller et al., 2000; Schiller and Schiller, 2001; Gasparini et al., 2004; Polsky et al., 2004; Losonczy and Magee, 2006; Nevian et al., 2007; Johnston and Narayanan, 2008; Losonczy et al., 2008; Larkum et al., 2009; Hay et al., 2011; Narayanan and Johnston, 2012; Major et al., 2013; Smith et al., 2013; Shai et al., 2015; Gidon et al., 2020). Until recently, the study of intracellular dendritic spikes *in vivo* from awake-behaving animals was not possible owing to the challenges associated with performing these recordings from thin dendrites while the animal moves around. (Smith et al., 2013) were able to achieve this difficult feat of performing patch clamp recording from the thin dendrites of the visual cortex pyramidal neurons *in vivo*. They showed that visual stimulation triggered regenerative local dendritic spikes (that were distinct from back-propagating action potentials) that enhanced the orientation selectivity.

Further, in 2017 Mayank Mehta’s group was serendipitously able to record membrane potential and spikes from cortical dendrites using tetrodes in awake behaving rats (Moore et al., 2017). They found that dendritic tips being thinner than the gaps between the tetrode bundles got caught in these gaps and were ensheathed by glial cells, enabling the recording of intracellular (quasi-in cell) dendritic voltage. Through this break-through, they demonstrated striking differences between dendritic and somatic spikes. For instance, the mean rate was found to be higher for dendritic spikes than for somatic spikes, indicating that not all dendritic spikes reach the cell body to yield axo-somatic action potentials (Golding and Spruston, 1998; Losonczy and Magee, 2006; Larkum et al., 2009; Lovett-Barron et al., 2012; Major et al., 2013; Palmer et al., 2014). Furthermore, far more spikes were generated in the dendrites than at the soma during slow wave sleep, providing further evidence for an observation that was made decades ago in anesthetized rats in the hippocampus as well using intracellular sharp electrodes (Kamondi et al., 1998a). Back then, it was discovered that large amplitude fast dendritic spikes coincided with CA1 sharp waves in the LFP and preceded spontaneously occurring dendritic Ca^2+^ spikes. While the cortical dendritic spikes recorded by (Moore et al., 2017) matched the features of a dendritic Na^+^ spike, in the same year another study found that slow dendritic calcium spikes were also clearly detectable from the cortical surface of rodents *in vivo* and in the LFP in response to sensory stimulation (Suzuki and Larkum, 2017). One of the first demonstrations that established the link between slow dendritic calcium spikes and ECoG recordings was by (Helmchen et al., 1999). In this study, (Helmchen et al., 1999) showed that, during whisker stimulation, complex spikes recorded intracellularly from distal dendrites and sharp waves in the ECoG were accompanied by large dendritic calcium transients. The highly active dendritic structure and the role of active dendritic channels in mediating spike initiation and propagation point to the need for accounting for dendritic supra-threshold ion channels and their currents in assessing LFPs.

Recently, another study discovered a new class of calcium-mediated dendritic action potentials (dCaAPs) in slices taken from surgically resected brain tissue of epilepsy patients (Gidon et al., 2020). The authors argue that dCaAPs are different from dendritic sodium spikes, calcium spikes, plateau potentials and NMDA spikes previously reported in rodents (Schiller et al., 1997; Helmchen et al., 1999; Larkum et al., 1999; Oakley et al., 2001; Larkum and Zhu, 2002; Waters et al., 2003; Milojkovic et al., 2005; Zhou et al., 2006a; Zhou et al., 2006b; Nevian et al., 2007; Major et al., 2008; Larkum et al., 2009; De Zeeuw et al., 2011; Moore et al., 2017). These dCaAPs were also observed in brain slices from tumor patients. Through computational analyses, the authors show that these dCaAPs enable dendrites to perform XOR operation, while sodium and NMDA spikes enable AND/OR logical operations at the soma and at tufts and basal dendrites respectively. This opens up interesting avenues for future exploration of the role of these various forms of suprathreshold events and suprathreshold conductances in dendritic computation and LFP signatures, both experimentally and computationally. As dendritic calcium spikes, plateau potentials, and NMDA spikes are relatively slow events, and are critically reliant on active dendritic components, their contributions to the low-frequency LFPs needs further analyses. This is especially important given the role of dendritic slow calcium spikes and plateau potentials in sensory-motor feedback, learning, perception, and plasticity (Takahashi and Magee, 2009; Lavzin et al., 2012; Xu et al., 2012; Larkum, 2013; Bittner et al., 2015; Manita et al., 2015; Takahashi et al., 2016; Bittner et al., 2017; Ranganathan et al., 2018; Aru et al., 2020; Doron et al., 2020; Magee and Grienberger, 2020; Roome and Kuhn, 2020; Takahashi et al., 2020; Bonnan et al., 2021). Thus, signatures of these slow dendritic events in the LFP could provide useful handles to analyses and interpretation of several physiological and behavioral out-comes, and constitutes an important future area for exploration.

Finally, in many scenarios the contribution of active dendritic conductances to LFPs is *driven* by afferent synaptic inputs and by the nonlinear somato-dendritic processing of these inputs. However, there are several neurons across different brain regions, including the cerebellum, suprachiasmatic nucleus, vestibular nuclei, and the dopaminergic midbrain structures, that are capable of spontaneously firing action potentials or bursts (Hausser et al., 2004; Welsh et al., 2010; Constantin et al., 2013; Hastings et al., 2018; Harvey et al., 2020; Otomo et al., 2020). Given that the ionic basis for such spontaneous activity is well understood in several of these neurons, LFPs in brain regions endowed with spontaneously firing neurons could manifest signatures that are independent of synaptic inputs, instead driven by ionic currents associated with spontaneous action potentials. Importantly, some of these neurons, including the cerebellar Purkinje cells (Hausser et al., 2004; De Zeeuw et al., 2011; Kitamura and Hausser, 2011; Roome and Kuhn, 2018, 2020), are endowed with elaborate dendritic trees and manifest slow dendritic spikes that are associated with spontaneous burst firing. In these neurons, the active dendritic contributions to relatively low-frequency components of the LFP could again be independent of synaptic inputs and could instead be driven by dendritic ionic currents. Thus, an important lacuna in the field pertains to the impact of active dendrites in spontaneously firing and bursting neurons on LFPs in brain regions endowed with such neurons.

### Subthreshold dendritic conductances and LFPs

While spikes inarguably provide immense information about neurophysiology, not all activity in neurons is suprathreshold. The underlying subthreshold membrane potential dynamics can vastly change neuronal output and information transfer. What mechanisms exist for a neuron to contribute to, process, and respond to such subthreshold voltage dynamics? The answer lies in the myriad of subthreshold active conductances expressed in the somato-dendritic compartments. In this section, we explore the roles of a few of these mechanisms, with illustrative examples involving different subthreshold ion channels, and argue for a strong impact of subthreshold dendritic conductances on LFPs.

#### A-type Potassium Channels, Astrocytes, and Calcium Waves

Voltage-gated transient *A*-type potassium channels, also referred to as “dendritic shock absorbers” (Yuste, 1997) tend to have almost 5-fold higher expression density in the dendrites than in the soma, and despite being subthreshold-active can in fact prevent suprathreshold dendritic spike initiation and propagation, thereby restraining large rapid dendritic depolarization (Hoffman et al., 1997; Migliore et al., 1999). Through morphologically realistic conductance based computational modelling it was shown that these dendritic *A*-type potassium channels can interact with the endoplasmic reticulum (ER) through inositol triphosphate receptors (InsP_3_R) and voltage-gated calcium channels, and can regulate the latency and temporal spread of calcium waves in dendrites (Ashhad and Narayanan, 2013). Large scale calcium waves have also been observed travelling through the astrocytic syncytium *in vivo* (Kuga et al., 2011; Sasaki et al., 2011; Ross, 2012; Ross and Manita, 2012). These astrocyte-mediated calcium waves have a role in synchonizing cortical activity as suggested by the inhibition of spontaneous cortical UP states upon chelation of calcium in cortical astrocytes (Poskanzer and Yuste, 2011). Astrocytic “glissandi” (the regenerative propagation of waves from cell to cell) are correlated with reduced infra-slow rhythms (<0.1–0.5 Hz) in the LFP (Hughes et al., 2011; Kuga et al., 2011).There is a strong phase-locking of interictal events and K-complexes to these infra-slow rhythms during sleep (Vanhatalo et al., 2004; Kang et al., 2005). This together with the fact that astrocytes can regulate the extracellular K^+^ concentration and modulate inhibitory synaptic activity highlights a significant role of astrocytic function and dysfunction in epilepsy and epileptiform activity (Kang et al., 1998; Vanhatalo et al., 2004; Kang et al., 2005; Coulter and Steinhauser, 2015; Nikolic et al., 2020).

Furthermore, astrocytes themselves express several voltage-gated ion channels and receptors, and can release calcium and neurotransmitter molecules (Verkhratsky and Steinhauser, 2000; Halassa and Haydon, 2010; Araque et al., 2014; Bazargani and Attwell, 2016; Ashhad and Narayanan, 2019). Through this gliotransmission, it has also been observed that astrocytes can induce large, long-lasting and slow excitatory potentials (SEPs) or dendritic plateau potentials in CA1 pyramidal neurons (Ashhad and Narayanan, 2016). The kinetics of these SEPs vary based on the location of stimulated astrocytes (close to proximal vs. distal apical dendritic compartments) and distinct NMDA receptor currents. Pharmacological analyses coupled with morphologically realistic conductance-based computational models have demonstrated that dendritically expressed hyperpolarization-activated cyclic-nucleotide–gated (HCN) and transient A-type potassium channels play critical roles in regulating the amplitude, kinetics, and compartmentalization of such SEPs (Ashhad and Narayanan, 2016, 2019). These studies highlight that while astroglia do not exhibit suprathreshold activity such as spikes, they can definitely regulate both LFPs and sub-/supra-threshold activity through interactions with dendritic subthreshold-active conductances and receptors under physiological and pathological conditions.

#### T-type Calcium Channels and NREM Sleep Spindles

*T*-type calcium channels and cation non-specific HCN channels are two other prominent subthreshold-active conductances that are highly expressed in proximal and distal dendrites of hippocampal and thalamo-cortical neurons, and can modulate intrinsic oscillations (McCormick and Pape, 1990; Leresche et al., 1991; Soltesz et al., 1991; Destexhe et al., 1993; Magee and Johnston, 1995; Destexhe et al., 1996; Johnston et al., 1996; Luthi and McCormick, 1998b, a; Magee, 1998, 1999; Hutcheon and Yarom, 2000; Santoro et al., 2000; Perez-Reyes, 2003; Crunelli et al., 2006; Narayanan and Johnston, 2012; Simms and Zamponi, 2014). In the thalamus, during the transition from wakefulness to sleep there is a reduction in the depolarizing tone exerted by afferents onto thalamocortical (TC) neurons and the nucleus reticularis thalami (nRt) (Huguenard and McCormick, 1992; McCormick, 1992a, b; McCormick and Huguenard, 1992; McCormick and von Krosigk, 1992; Steriade and Timofeev, 2003). This is associated with a prolonged hyperpolarization of the majority of the recipient neurons into membrane potentials where activation and inactivation of *T* channels is possible (Roy et al., 1984; Steriade and Timofeev, 2003; Fuentealba et al., 2004). Once de-inactivated by hyperpolarization, *T*-type calcium channels mediate low-threshold Ca^2+^ spikes, which in turn trigger a burst of action potentials during different stages of NREM sleep (Deschenes et al., 1982; Llinas and Jahnsen, 1982; Deschenes et al., 1984; Jahnsen and Llinas, 1984a, c, b; Steriade and Deschenes, 1984; Domich et al., 1986; Llinas and Steriade, 2006). Owing to this oscillatory property and burst synchronization of the thalamocortical circuit by *T*-type calcium channels, they are regarded as the major pacemakers responsible for rhythmogenesis of thalamocortical sleep spindles during NREM sleep (McCormick and Bal, 1997; Cueni et al., 2008; Astori et al., 2011; Pellegrini et al., 2016).

Further, it has been shown that in the GABAergic nRt neurons, the triad of dendritic small conductance type K^+^ (*SK2*) channels, sarco/endoplasmic reticulum Ca^2+^-ATPase (SERCA) pumps, and *T*-type calcium channels regulates oscillatory dynamics related to sleep. Specifically, the oscillatory bursting is initiated via selective activation of dendritic SK2 channels while the dampening of the oscillation is mediated through Ca^2+^ uptake by SERCA pumps and cumulative *T*-type calcium channel inactivation (Cueni et al., 2008). The relevance of SK2 channels was highlighted through studies on *SK2*^-/-^ mice that lack cellular oscillations, show a large (>3 fold) reduction in low-frequency rhythms during NREM sleep, and have disrupted sleep (Cueni et al., 2008). Deletion of Ca_V_3.2 and Ca_V_3.3 *T*-type Ca^2+^ channels has also been shown to suppress sleep spindle rhythmogenesis in mice (Pellegrini et al., 2016) and the absence of Ca_V_3.3 channels prevents oscillatory bursting in the theta-frequency range in nRt cells (Astori et al., 2011). However, sleep spindles are not altered in Ca_V_3.1^−/−^ mice during natural NREM sleep (Lee et al., 2013). This highlights the role of specific ion-channel sub-types/isoforms in mediating biological rhythms and perhaps that in knockouts such as these, a compensatory mechanism is employed by the neurons to sustain oscillatory response dynamics. It is possible that this compensatory mechanism could be mediated through upregulation of HCN channels, which have also been shown to regulate sleep spindles (McCormick et al., 2015). In fact, based on observations in humans, a model for coordination between cortical UP-DOWN states and thalamic spindles during NREM sleep has been proposed which involves both *T*-type Ca^2+^ channels and HCN channels (Mak-McCully et al., 2017).

#### HCN Channels and Theta Oscillations

Unlike most ion channels, HCN channels have a unique property, as the name suggests, of activating upon hyperpolarization. In pyramidal neurons, they are active at the resting membrane potential, have slow activation and deactivation kinetics and their expression density increases from soma to dendrites, being highest in the distal apical dendrites (Magee, 1998; Williams and Stuart, 2000; Lorincz et al., 2002; Migliore and Shepherd, 2002; Kole et al., 2006; Narayanan and Johnston, 2007; He et al., 2014). They regulate neuronal excitability by reducing the temporal summation of EPSPs at the soma (Magee, 1998, 1999, 2000; Williams and Stuart, 2000; Narayanan and Johnston, 2007). As a confluence of these properties, they oppose changes to membrane potential, reduce the excitability of dendrites by reducing their input resistance, confer an increasing gradient of dendritic theta-frequency resonance, contribute an inductive component to the input impedance, and mediate the location- and activity-dependence of the intrinsic phase response (Hutcheon and Yarom, 2000; Ulrich, 2002; Narayanan and Johnston, 2008, 2012). Their somatodendritic expression acts as a gradient of inductance which helps resolve the locationdependent temporal differences in dendritic inputs by synchronizing the theta- and gamma- frequency inputs to a common synchronization frequency (transfer resonance frequency) at the soma (Vaidya and Johnston, 2013). This synchronization frequency happens to be in the theta-frequency range, and is invariant to input location. HCN channels can also affect spike initiation dynamics and have the capability of reducing the coincidence detection window to temporal ranges within the low and high gamma frequency ranges at lower and higher conductance values, respectively (Das and Narayanan, 2014, 2015, 2017; Das et al., 2017).

**Fig. 2:**
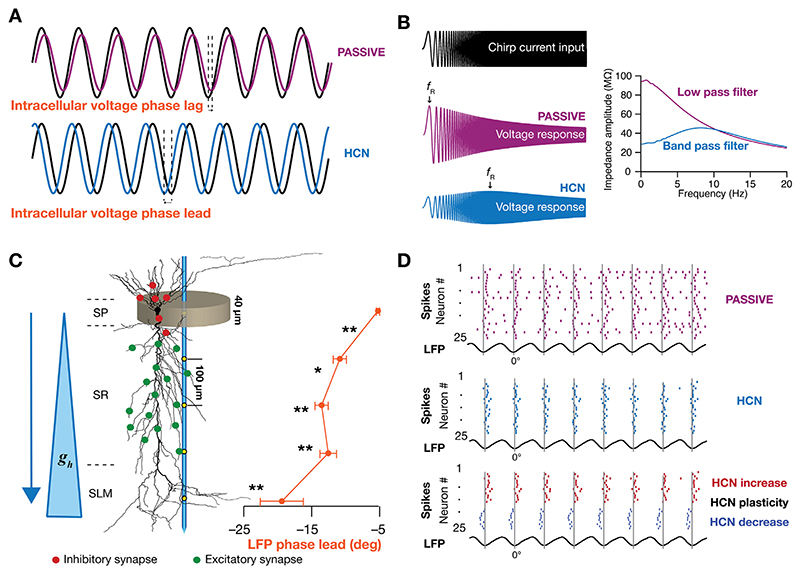
HCN channels regulate intracellular and extracellular voltage responses to theta frequency inputs. **(A)** Voltage response of a passive neuronal compartment (top) and of a neuronal compartment expressing HCN channels (bottom) to a theta-frequency sinusoidal current injection (black traces) for 1 s. Notice that the passive voltage response shows a phase lag with respect to the input current and a reduction in amplitude while the voltage response in the presence of HCN channels shows a corresponding phase lead (Narayanan and Johnston, 2008). **(B)** *Left top*: A chirp stimulus, constant amplitude sinusoidal current linearly increasing in frequency from 0 to 20 Hz in 20 s. *Left middle*: Passive voltage response to the chirp stimulus showing low-pass filtering. *Left bottom*: Voltage response in the presence of HCN channels showing a band-pass response with the highest amplitude at its resonance frequency (*f*_R_). *Right*: Illustrative impedance amplitude profiles show a low-pass response for the passive compartment and a band-pass response in the presence of HCN channels, with *f*_R_ in the theta-band. Such low-pass and band-pass responses are also reflected in the LFP power spectral density in the absence and presence of HCN channels, respectively (see (Ness et al., 2016, 2018)) **(C)** *Left*: A morphologically realistic CA1 pyramidal neuron model representing a population of neurons in a cylindrical neuropil, with inhibitory synapses targeting the perisomatic regions and excitatory synapses targeting the dendrites in the SR. An experimentally constrained HCN channel gradient was introduced in the model accounting for an increase in HCN conductance (*g_h_*) with distance from the soma. A single electrode with seven recording sites, located at the center of the cylindrical neuropil spanned all strata of the CA1. *Right*: Lead in the LFP phase introduced by the presence of HCN channels increases with HCN conductance along the somato-apical axis ((Sinha and Narayanan, 2015); *: *p* < 0.05; **: *p* < 0.005; Wilcoxon signed rank test). **(D)** *Top*: Raster plots for 25 passive model neurons with low spike-phase coherence with respect to theta frequency LFP. *Middle*: Same as top but with a gradient of HCN channels introduced in the model shows enhancement of spike-phase coherence and spike theta-phase preference. *Bottom*: Same as middle but illustrating the impact of bidirectional HCN plasticity on reconfiguration of cell assemblies. SP: *stratum pyramidale*, SR: *stratum radiatum*, SLM: *stratum lacunosum moleculare*.

When an oscillating current in the theta-frequency range is injected in neurons expressing HCN channels, it is observed that the intracellular voltage response leads in phase with respect to the current (Ulrich, 2002; Narayanan and Johnston, 2008) (Fig. 2A), a property of an inductor, and that this phase lead manifests in the theta-frequency range. A morphologically realistic conductance-based experimentally constrained computational model employing a forward modeling scheme for LFPs showed that this phase lead mediated by HCN channels is in fact reflected even in the theta-frequency LFP in the hippocampus (Sinha and Narayanan, 2015). Given that the expression density of HCN channel increases with distance from the soma towards the apical dendrites, it was shown that the phase lead also increases along the somato-apical axis (Fig. 2B). The ability of HCN channels to introduce an inductive phase lead in intracellular voltage responses to theta-modulated synaptic currents played a significant role in altering LFP and spike phases.

CA1 and CA3 pyramidal neurons tend to show phase preference with the maximum firing probability at the trough of a theta cycle (Csicsvari et al., 1999; Klausberger and Somogyi, 2008). (Sinha and Narayanan, 2015) showed that HCN channels can regulate this spike theta-phase preference of individual neurons and at a population level HCN channels enable formation of cell assemblies by enhancing spike thetaphase coherence (Fig. 2D). It was also demonstrated that a single neuron could shift its spike-phase preference through HCN channel plasticity. To our knowledge, this was the first direct evidence of a subthreshold conductance influencing the ongoing LFP and also the spike-phase response of the neurons in the hippocampus. The study further demonstrated a critical role of the phase of inhibitory input arrival with reference to excitatory inputs in altering both the *stratum pyramidale* LFP and associated spike phases, but not spike phase coherence. Thus, there exists a critical role of HCN channels and synaptic receptors in phase-coding schemas and in the formation and dynamic reconfiguration of neuronal cell assemblies. Following this modeling study, two other modeling studies demonstrated the strong influence of subthreshold HCN channels, this time, on cortical LFPs (Ness et al., 2016, 2018). They showed that a gradient of HCN channels can induce theta-frequency resonance in the LFP signal (Fig. 2C).

Together, these studies present a clear case for the incorporation of subthreshold-activated ion channels, their distribution across the cell membrane, and their plasticity into the computation of LFPs (Sinha and Narayanan, 2015; Ness et al., 2016, 2018). They also strongly highlight how biophysically and morphologically realistic experimentally-constrained computational models can advance our mechanistic understanding of neuronal function in generating neural codes, LFPs, and behavior.

### Morphology of active dendrites and LFPs

Dendritic morphology critically regulates neuronal physiology. Although it was traditionally assumed that dendritic arborization merely provides larger surface area to accommodate more synapses, it is clear that the arborization allows for far more functional specializations than acting as a simple funnel for information through a large number of synaptic inputs (Poirazi and Mel, 2001; Poirazi et al., 2003; London and Hausser, 2005; Johnston and Narayanan, 2008; Narayanan and Johnston, 2012; Poirazi and Papoutsi, 2020). The presence of complex dendritic arborization in conjunction with active-dendritic conductances mediates segregation and compartmentalization of afferent inputs, thereby allowing for location dependence in input processing and filtering, dendritic spike initiation, coincidence detection, and even specialized dendro-dendritic communication in certain synapses (Rall and Shepherd, 1968; Golding and Spruston, 1998; Schiller et al., 2000; Schiller and Schiller, 2001; Gasparini et al., 2004; Polsky et al., 2004; Losonczy and Magee, 2006; Narayanan and Johnston, 2007; Nevian et al., 2007; Losonczy et al., 2008; Narayanan and Johnston, 2008; Larkum et al., 2009; Gidon et al., 2020). Dendritic morphology plays a critical role in regulating neural excitability, firing patterns, coincidence detection, and the expression of functional gradients, even in the presence of gradients in ion channel expression (Mainen and Sejnowski, 1996; Vetter et al., 2001; Krichmar et al., 2002; van Ooyen et al., 2002; Cannon et al., 2010; Narayanan and Chattarji, 2010; Ferrante et al., 2013; Dhupia et al., 2015; Ostojic et al., 2015; Das et al., 2017). Changes in any of these functional properties significantly alter the transmembrane currents through ion channels and receptors on the somato-dendritic arbor, thus altering the recorded LFPs at different electrode locations. Thus, the structural organization of the dendritic tree and the arrangement of dendritic arbors of different neurons in the subregion (*e.g*., open field organization in hippocampus and cerebellum *vs*. random organization in several other brain regions) should be accounted for as critical regulators of the strength and polarity of field potentials (Johnston and Wu, 1995; Linden et al., 2011; Buzsaki et al., 2012; Einevoll et al., 2013). The arborization of axons and their localization on the dendritic arbor also play essential roles in regulating the spread and shape of unitary LFPs (Glickfeld et al., 2009; Bazelot et al., 2010; Telenczuk et al., 2020b). Databases of neural morphologies (Ascoli et al., 2007; Gouwens et al., 2019) and algorithms for dendritic remodeling (Narayanan et al., 2005; Koene et al., 2009; Cuntz et al., 2010; Narayanan and Chattarji, 2010; Bozelos et al., 2015; Dhupia et al., 2015; Beining et al., 2017), coupled with biophysically realistic computational modeling, could be employed to systematically assess the impact of active dendritic morphology on LFPs.

## Gradients, Heterogeneities and Degeneracy

### Gradients and heterogeneities in ion-channel expression and intrinsic properties

Neurons manifest gradients in the spatial expression profiles of active ion-channel conductances. There are systematic lines of evidence for the existence of such gradients *within* individual neurons (Migliore and Shepherd, 2002; Johnston and Narayanan, 2008; Nusser, 2009; Narayanan and Johnston, 2012), as well as *across* neurons spanning specific anatomical axes (Giocomo et al., 2007; Igarashi et al., 2014; Lee et al., 2014; Malik et al., 2016; Maroso et al., 2016; Malik and Johnston, 2017; Sun et al., 2017; Valero and de la Prida, 2018; Cembrowski and Spruston, 2019; Navas-Olive et al., 2020; Pastoll et al., 2020). Gradients in ionchannel expression profile *within* a single neuron, along its somatodendritic axis, yield functional maps of neuronal properties. From the standpoint of LFPs, these gradients imply location-dependent contributions of individual ion channels to extracellular field potentials. For instance, hippocampal CA1 pyramidal neurons manifest a gradient in HCN ion channels, with higher expression at distal dendritic locations (Magee, 1998; Lorincz et al., 2002). A primary implication for the expression of this gradient is an increase in the amount of HCN-channel mediated transmembrane currents in distal dendritic locations. However, as HCN channels reduce the excitability of neurons (Gasparini and DiFrancesco, 1997; Magee, 1998; Narayanan and Johnston, 2007), the HCN-channel gradient also implies that the voltage response to a given synaptic current is lower in distal locations. This reduced voltage deflection, in turn, alters the driving forces for the different ion channels and receptors expressed at that location, thereby altering the transmembrane currents through these distinct ion channels and receptors. In addition, the reduced voltage responses also alter the capacitive current in a distance dependent manner as the rate of change in voltage responses is altered by the slow kinetics of HCN channels. The impact of the expression of the HCN channel gradient, therefore, is the cumulative and synergistic impact of changes to all currents, and depends on the patterns of synaptic activation, the ultra-structural morphology, and the relative expression profiles of individual receptors and channels (Sinha and Narayanan, 2015; Ness et al., 2016, 2018; Navas-Olive et al., 2020).

If a computational approach is employed to assess the impact of intraneuronal ion-channel gradients on LFP generation, it is essential to account for neuronal morphology and specific ion-channel gradients. Importantly, these ion-channel gradients should be quantitatively matched with signature somato-dendritic functional maps of that specific neuron (Narayanan and Johnston, 2012; Rathour and Narayanan, 2014; Dhupia et al., 2015). For instance, in assessing the impact of HCN-channel gradients on theta-frequency LFPs, (Sinha and Narayanan, 2015) matched functional maps on local resonance, transfer resonance, and input resistance across the somatodendritic axis before employing the model for computing LFP. (Sinha and Narayanan, 2015) found that the impact of HCN channel gradient on theta-frequency LFP was location-dependent, with changes in LFP phase. Without setting ion-channel gradients and conductances to match electrophysiologically determined functional maps and other physiological properties, the outcomes of such analyses will result in conclusions that overestimate or underestimate the role of a specific ion channel (and its gradients) on the LFP. As LFP is an outcome of distance-dependent summation of different ionic currents emanating from different locations, it is essential that the gradients in all ion channel properties are carefully matched with respective experimental outcomes and the specific patterns of synaptic activity (Linden et al., 2011; Leski et al., 2013) are accounted for in assessing the impact of active dendritic gradients on LFP.

Apart from intraneuronal gradients in ion channel properties, there are also systematic gradients in ion channel expression and intrinsic properties across neurons of the same subtype in different parts of the same brain region. For instance, there are gradients in CA1 pyramidal neuron ion-channel expression and intrinsic properties along the dorso-ventral, proximodistal and deep-superficial axes of the hippocampus (Igarashi et al., 2014; Lee et al., 2014; Malik et al., 2016; Maroso et al., 2016; Malik and Johnston, 2017; Sun et al., 2017; Valero and de la Prida, 2018; Cembrowski and Spruston, 2019; Navas-Olive et al., 2020). An important lacuna in the field pertains to the evaluation of the impact of such inter-neuronal gradients in somato-dendritic properties on LFPs for different patterns of synaptic activation. A systematic electrophysiological and computational evaluation spanning each of these distinct anatomical axes, including the somatodendritic axis of neurons in each subregion, is essential for understanding the impact of intra-neuronal and interneuronal gradients in active dendritic properties on the location-dependence of LFP emergence.

Finally, even within a given subregion, neurons of the same subtype are not identical in terms of their ion channel distributions or their intrinsic properties. Instead, there is considerable cell-to-cell heterogeneity in ion channels and cellular properties even within the same cell types of the same brain region (Rathour and Narayanan, 2014; Malik et al., 2016; Rathour and Narayanan, 2019; Mishra and Narayanan, 2020; Pastoll et al., 2020). In addition, there is animal-to-animal heterogeneity in how gradients manifest along a specified anatomical axis (Pastoll et al., 2020). It is essential that these heterogeneities are not ignored by assuming networks to be composed of homogenous neuronal populations, but are explicitly characterized from specific brain regions and are accounted for in experimental analyses and computational simulations. Without such explicit incorporation, the differential ionic contributions from different neurons, despite them receiving identical input patterns of activity (Mishra and Narayanan, 2021) will be neglected.

### Degeneracy in the emergence of characteristic neuronal properties and LFPs

It is now well established that neuronal systems such as single neurons and networks of neurons can perform their characteristic functions despite heterogeneities in underlying system parameters and hence can be quite robust to perturbations (Prinz et al., 2004; Marder, 2011; Marder and Taylor, 2011; Rathour and Narayanan, 2012, 2014; Drion et al., 2015; Mishra and Narayanan, 2019; Rathour and Narayanan, 2019; Goaillard and Marder, 2021). This is an example of degeneracy in the biological context where degeneracy is described as the ability of structurally disparate elements to perform the same function (Tononi et al., 1999; Edelman and Gally, 2001; Stelling et al., 2004; Rathour and Narayanan, 2019). While neurons of the same kind (*e.g.,* pyramidal neurons) manifest characteristic functional outcomes, these neurons are typically morphologically heterogeneous with intricate dendritic arbors. Within these delicate arbors, lies another layer of heterogeneity: the diverse expression profiles of intrinsic properties such as ion channels and receptors. Depending on their location, two dendritic branches even of similar length can express completely different sets of ion channels or their expression profiles in terms of their density could be different. There could even be different subunits (main or auxiliary) of the same ion channel expressed at different locations. On top of that, depending on their location in the morphological tree, dendritic branches can receive completely distinct synaptic inputs, leading to a differential expression of and response from synaptic and extra-synaptic receptors. Furthermore, if the study involves looking at the mechanisms underlying changes in LFPs due to incoming synaptic inputs, there exists the additional involvement of not only activity-dependent synaptic plasticity but also potentially concurrent intrinsic and structural plasticity. Thus, it is highly important that any computational study that is trying to model LFPs for gaining mechanistic insights takes care of introducing the appropriate level of abstraction and heterogeneity to incorporate the underlying variability in the system. This will help avoid incorrectly attributing a single cause for an extracellular phenomenon when there could be multiple underlying causes in a complex neural system generating the LFP.

Finally, whereas these analyses point to the expression of parametric degeneracy in the emergence of single-neuron and network functions, there are strong lines of evidence for the manifestation of degeneracy at the level of LFP rhythmogenesis, whereby LFP rhythms can be generated through disparate mechanisms (Rathour and Narayanan, 2019). It is critical that these disparate routes towards generating signature LFP patterns and rhythms are identified, employing experimental and computational techniques, to exercise extreme caution in making one-to-one relationships between underlying parameters and specific characteristics of LFP recordings (Rathour and Narayanan, 2019).

### Accounting for gradients, heterogeneities and degeneracy in models of LFP

#### Multi-Parametric, Multi-Objective Stochastic Search Algorithms

**Fig. 3:**
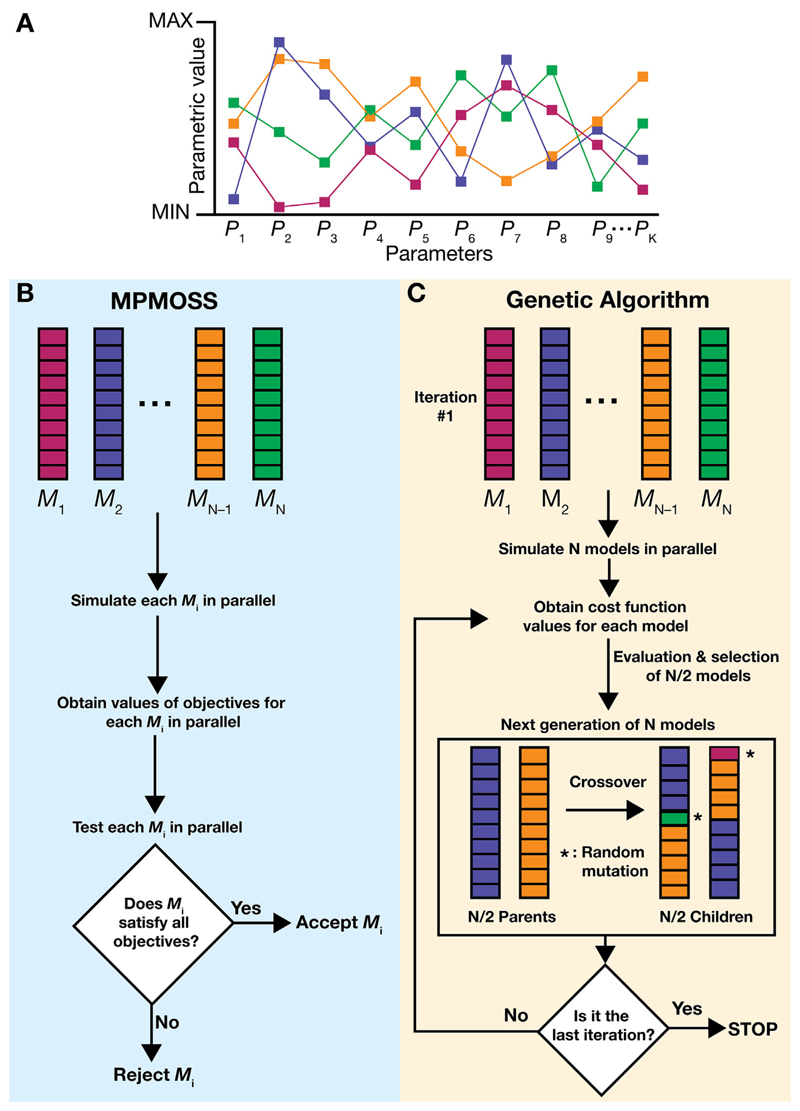
Accounting for heterogeneity and degeneracy in computational models **(A)** Parameter values (*P*_i_) are picked randomly from their respective experimental ranges. A set of parameters constitutes a model/individual, *M*_i_. **(B)** MPMOSS algorithm. **(C)** Genetic algorithm (GA). Note that while MPMOSS is a highly parallelized algorithm, GA is by design iterative and spans several generations. There are several variants of the generic GA depicted here, with slight modifications to each step suited for different purposes.

A method that is commonly employed to generate biophysically and physiologically realistic neuron models that account for heterogeneities and degeneracy is the multi-parametric, multi-objective, stochastic search (MPMOSS) (Foster et al., 1993; Taylor et al., 2009; Marder and Taylor, 2011; Rathour and Narayanan, 2012, 2014; Basak and Narayanan, 2018; Mittal and Narayanan, 2018; Mishra and Narayanan, 2019; Jain and Narayanan, 2020; Seenivasan and Narayanan, 2020). The generic algorithm for a specific morphology for a given subtype of neuron in a given subregion involves the following steps (Fig. 3):

1. Multiple Parameters (MP): Identify the crucial parameters of the neuron. Ideally, these parameters span all passive and active properties of the neuron under consideration, because physiology emerges as a consequence of intricate interactions among all these properties. However, this could also be a subset of parameters, depending on the specific question in hand. The crucial parameters span intrinsic passive (*e.g.,* axial resistance, the leak channel conductance and the specific membrane capacitance, and their location dependent gradients), and active (*e.g.,* conductance, gating properties, (de)activation/inactivation kinetics, reversal potential and location-dependent gradients) properties.
2. Define the ranges of these parameters from physiological observations for the specific neuronal subtype. Do not employ average or other summary statistics. Consider the range of individual parameters to encompass the entire span of experimental measurements from individual cells, thereby accounting for cell-to-cell variability.
3. Multiple Objectives (MO): Define the set of objectives that this neuronal model must fulfill based on experimental measurements. For our example, these could be constraints on the resting membrane potential, the input resistance, and the firing rate response to a specific stimulus for this neuronal type. Define lower and upper bounds for each of these measurements from experiments on the specific neuronal subtype. Note that we are not employing summary statistics here as well, but are accounting for the heterogeneities in characteristic neural measurements in that specific region.
4. Stochastic Search (SS):
  - For each parameter defined in step 1, randomly pick one value within its specified range defined in step 2. This set of parametric values will then be used to simulate one model neuron.
  - Perform step 4a multiple times to generate unique combinations of parametric values and thus generate a large number of model neurons.
  - Search for valid models: Perform the search by testing each of these models for their validity by assessing whether they satisfy the multiple objectives in Step 3 or not. Declare models that have *all* their measurements within the bounds defined in Step 3 to be valid models.

#### Genetic Algorithms

In a comparative survey of automated parameter-search methods for compartmental neural models, genetic algorithms (GA) outperformed other methods for both simple and complex multicompartmental models (Vanier and Bower, 1999). GA are iterative optimization algorithms inspired from mechanisms of Darwinian evolution. In these, each morphologically realistic multicompartmental model neuron is considered as an *individual* from a genetically diverse *population*. Various intrinsic, synaptic, and morphological parameters define an individual (Fig. 3). In the first iteration, a random population is usually generated.

To search for optimal parameters (solutions) for a population of such individuals, first *target functions* are defined based on experimental data. These could be distance-dependent/independent intrinsic properties such as input resistance, resting membrane potential, resonance frequency, and axial resistance and/or synaptic properties such as excitatory/inhibitory postsynaptic potentials. The values of these target functions are computed for each individual. Then errors (square of difference) are computed between the experimentally and computationally obtained target function values for each individual. These errors help define the fitness of the individuals, towards discerning whether they lie within the experimentally observed variability or not. All individuals are sorted based on their *cost function* (such as the sum of all the errors of a given individual).

From this list, pairs of individuals are randomly chosen and the individuals with the lower cost function (*i.e.*, they are closer to the experimental observations) are selected for *breeding* the next *generation* (iteration). Pairs of these selected individuals act as *parents* and undergo a *crossover* (exchange of parameters) to produce pairs of children with *genetic diversity*. These children may also undergo random *mutations* (probabilistic changes in the current values of parameters by a small amount that diminishes with each iteration).

This process of evaluation, selection, breeding/ crossover, mutation and new solution generation is continued until either a set bound on the number of iterations has been reached or if a certain criterion for population fitness (such as optimal matches between experimental results and model performance) has been achieved. Several variants of this algorithm have been successfully used in generating a diverse set of model neurons that match experimental observations, and in some cases have been released as open-source software as well (Keren et al., 2005; Druckmann et al., 2007; Menon et al., 2009; Bahl et al., 2012; Friedrich et al., 2014; Van Geit et al., 2016; Neymotin et al., 2017; Gouwens et al., 2018; Navas-Olive et al., 2020).

#### Analyzing and Utilizing the Valid Model Population

As all models (obtained either through MPMOSS or GA) that are valid manifest signature physiological properties, with biophysically matched parametric ranges, this model population can then be employed to assess parametric dependencies in the single-neuron population (Foster et al., 1993; Taylor et al., 2009; Marder and Taylor, 2011; Rathour and Narayanan, 2012, 2014; Basak and Narayanan, 2018; Mittal and Narayanan, 2018; Mishra and Narayanan, 2019; Jain and Narayanan, 2020; Navas-Olive et al., 2020; Seenivasan and Narayanan, 2020) or to construct heterogeneous network models that are representative of the specific brain region (Prinz et al., 2004; Mishra and Narayanan, 2019, 2021). The same algorithm must be repeated for different morphologies when accounting for heterogeneities in morphologies in the same neuronal subtype (Basak and Narayanan, 2020; Navas-Olive et al., 2020). NeuroMorpho is a great database of neuronal morphologies (Ascoli et al., 2007) and the Allen Cell Types Database (Gouwens et al., 2019) is a very useful database which provides not only neuronal morphologies, with characterization of electro-physiological properties and gene expression in individual neurons, but also a variety of models at different levels of resolution.

The MPMOSS and GA algorithms could be effectively employed for imposing signature intraneuronal functional maps on distinct neuronal morphologies and signature neuronal responses to different patterns of afferent inputs (Rathour and Narayanan, 2014; Basak and Narayanan, 2018, 2020). In addition, these algorithms could be employed to study inter-neuronal gradients (*e.g*., dorso-ventral), by altering the parametric and objective/target distributions employed in the algorithm to experimentally match specific subregions. Importantly, independent MPMOSS or GA simulations could be performed to generate distinct populations of models for each of the different neuronal subtypes in a given brain region (*e.g*., principal neurons and interneurons), to explicitly account for characteristic ion-channel properties and intrinsic measurements of each different subtype (Rathour and Narayanan, 2012, 2014; Mittal and Narayanan, 2018; Mishra and Narayanan, 2019; Jain and Narayanan, 2020). Finally, as these algorithms yield models with disparate parametric combinations for matching signature functional outcomes (constituting the manifestation of degeneracy) in any given subregion, they also provide the substrate for accounting for cell-to-cell heterogeneity in network models (Prinz et al., 2004; Mishra and Narayanan, 2019; Navas-Olive et al., 2020; Mishra and Narayanan, 2021). Thus, employing these algorithms for generating a population of models for different subregions constitutes an ideal way to construct heterogeneous networks that account for intra- and inter-neuronal gradients and heterogeneities in ion-channel and intrinsic properties of neuronal subtypes. Such physiologically matched heterogeneous networks should then be employed to assess the impact of active dendritic contributions to LFPs under different synaptic activation patterns.

Within this framework, the impact of individual ion channel subtypes on LFPs could be efficiently analyzed employing the virtual knockout framework (Rathour and Narayanan, 2014; Anirudhan and Narayanan, 2015; Sinha and Narayanan, 2015; Mukunda and Narayanan, 2017; Basak and Narayanan, 2018, 2020; Jain and Narayanan, 2020; Mishra and Narayanan, 2021). In implementing this, the conductance of individual ion channel subtypes in each of the several heterogeneous neurons (of a selected subtype) in the network is set to zero. The LFPs are computed for the two networks, one where the specific ion channel subtype is intact and another where the respective conductance values are set to zero for all neurons of the same subtype, with identical synaptic inputs. Comparison of LFPs in the two networks now provides an estimate of the specific contributions of an ion channel subtype on LFPs (*e.g*., (Sinha and Narayanan, 2015) for HCN-channel contribution to theta-frequency LFPs). This process could then be repeated for each of the different ion channel subtypes in the different neuronal subtypes to assess their contributions to LFPs at different locations. Together, the MPMOSS and GA algorithms are powerful tools for assessing active dendritic contributions to LFP, while accounting for all physiological constraints including heterogeneities, gradients and degeneracy.

## Neuromodulation, Plasticity and Neurological Disorders

The contributions of active dendritic conductances to LFPs are not static, but change in response to neuromodulation, short- and long-term plasticity and pathological insults.

### Neuromodulatory impact on active dendrites and LFP

Neuromodulation offers an ideal substrate for reconfiguring functional connectivity in neural circuits towards achieving behavioral context-dependent processing of sensory stimuli (Getting, 1989; Marder and Thirumalai, 2002; Lee and Dan, 2012; Marder, 2012; Bargmann and Marder, 2013; Marder et al., 2014; McCormick et al., 2020). Neuromodulators typically act through receptors expressed on the cellular surface. They can alter intrinsic properties and synaptic efficacy either directly or through activation of downstream signaling cascades. Within an active dendritic framework, such neuromodulatory action translates to changes in the transmembrane currents through dendritic ion channels and receptors (Hoffman and Johnston, 1999; Heckman et al., 2003; Rosenkranz and Johnston, 2006, 2007; Dembrow et al., 2010; Dembrow and Johnston, 2014; Santello and Nevian, 2015; Labarrera et al., 2018; Payeur et al., 2019; Williams and Fletcher, 2019; Yaeger et al., 2019), thereby pointing to a direct neuro-modulatory impact on recorded LFPs. Thus, it is critical that experimental analyses and computational frame-works on LFPs account for neuromodulatory reconfiguration of neural circuits and state-dependent changes to constitutive components of all cell types. Future experimental and computational studies should systematically map the location-dependent impact of different neuromodulatory inputs, impinging on disparate neural circuits with distinct spatiotemporal patterns, on the emergence of LFPs.

### Short- and long-term plasticity of intrinsic and synaptic properties

The brain is a highly plastic organ. The continual changes in brain structure and function span all of its components, with a wide variety of time scales over which such changes occur. Whereas changes involving short-term synaptic plasticity (Zucker and Regehr, 2002; Regehr, 2012) and conformational dynamics of molecules, including ion channels and receptors (Johnston and Wu, 1995; Dingledine et al., 1999; Hille, 2001), are on the faster time scale, there are other forms of plasticity that evolve over slower time scales (Kim and Linden, 2007; Gjorgjieva et al., 2016; Turrigiano, 2017; Zenke et al., 2017; Ma et al., 2019). These changes imply that the transmembrane currents through active dendritic receptors and channels would be subjected to long- and short-term changes. For instance, for the same burst of presynaptic activation, a facilitating synapse would manifest progressively increasing postsynaptic responses whereas a depressing synapse would yield progressively reduced responses. Thus, it is important to not treat synapses as linear time invariant structures (Tsodyks and Markram, 1997). Importantly, there are gradients in short-term plasticity profiles across the somato-dendritic axis (Jensen et al., 2021), implying that the profile of transmembrane currents and dendritic voltage responses are critically reliant on the specific short-term profile at individual dendritic locations. Similarly, there are established gradients in ion channel kinetics that regulate different aspects of dendritic physiology. For instance, in CA1 pyramidal neurons, the recovery rate of somatic sodium channels from inactivation is faster for somatic sodium channels compared to their dendritic counterparts. These kinetic differences translate to differences in the amplitudes of dendritic backpropagating action potentials within a train of action potentials (Colbert et al., 1997). These observations imply that the transmembrane currents through the several voltage-gated ion channels along the dendritic arbor respond differentially in response to the same backpropagating action potentials, depending on their dendritic location. Thus, short-term plasticity of synaptic and intrinsic properties, along with somato-dendritic gradients in these plasticity profiles should be systematically accounted for in analyzing the LFP profiles and their dependencies on active dendritic structures.

Long-term forms of plasticity that are involved in learning, memory and stability also span all components of active dendritic structures (Frick and Johnston, 2005; Magee and Johnston, 2005; Kim and Linden, 2007; Johnston and Narayanan, 2008; Narayanan and Johnston, 2012). In addition, synaptic and dendritic intrinsic properties could be altered in the short- and long-term by transmitter and messenger molecules released by the dendrites as well as adjacent glia (Regehr et al., 2009; Araque et al., 2014; Ashhad and Narayanan, 2016; Maroso et al., 2016; Ashhad and Narayanan, 2019). It should be noted that although these forms of plasticity might specifically target one specific ion channel or receptor, the consequent changes in transmembrane currents would span a larger swath of molecules owing to structural or functional interactions. For instance, an increase in the density of a synaptic receptor molecule would alter the postsynaptic voltage deflection, which in turn would change the voltage-dependent opening and driving forces of all ion channels and receptors that are affected by this voltage deflection. Thus, in assessing the impact of behaviorally induced changes on LFPs, changes in all transmembrane currents introduced by these disparate forms of plasticity should be systematically accounted for in a location-dependent manner for the different cells in the circuit. In assessing the impact of bidirectional long-term plasticity in HCN channels (Fan et al., 2005; Brager and Johnston, 2007; Narayanan and Johnston, 2007) on theta-frequency LFPs in hippocampal CA1, (Sinha and Narayanan, 2015) first quantitatively matched cellular plasticity in physiological measurements across the dendritic arbor. Specifically, (Sinha and Narayanan, 2015) matched global changes in input resistance and resonance frequency obtained from electrophysiological recordings (Fan et al., 2005; Brager and Johnston, 2007; Narayanan and Johnston, 2007) for bidirectional HCN-channel plasticity. Employing these models that underwent biophysically realistic plasticity, (Sinha and Narayanan, 2015) showed that graded changes in either HCN conductance or its half-maximal activation voltage resulted in graded changes in phases of theta-frequency LFP and associated spike (Fig. 2D).

### Neurological disorders, active dendrites, and LFP

Pathological conditions alter active dendritic properties. Ion channel dysfunction, referred to as channelopathy, is associated with several neurological disorders. Careful experimentation assessing ion channels involved in dendritic information processing has revealed dramatic pathological changes in their composition and densities (Kullmann, 2002; Bernard et al., 2004; Kullmann and Waxman, 2010; Remy et al., 2010; Terzic and Perez-Terzic, 2010; Emoto, 2011; Poolos and Johnston, 2012; Lerche et al., 2013; Brager and Johnston, 2014; Zhang et al., 2014; Johnston et al., 2016; Cook et al., 2021). These changes have been shown to be cell-type dependent, with the same ion channel subtype undergoing changes in opposite directions in different cell types with reference to the same pathological condition (Lee et al., 2011; Brager et al., 2012; Lee and Jan, 2012; Routh et al., 2013; Brager and Johnston, 2014; Zhang et al., 2014; Kalmbach et al., 2015; Routh et al., 2017; Siegel et al., 2017; Brandalise et al., 2020). In addition to channelopathies, neurological disorders alter several aspects of brain structure and function, including connectivity patterns, dendritic morphology and glial physiology (Schauwecker and McNeill, 1996; Maragakis and Rothstein, 2006; McEwen, 2007; Sofroniew and Vinters, 2010; Chattarji et al., 2015; Coulter and Steinhauser, 2015; Khakh and Sofroniew, 2015; Phatnani and Maniatis, 2015; Patel et al., 2019). As active dendritic function, and consequently the transmembrane currents across the different ion channels and receptors are critically regulated by each of these distinct parameters, it is essential that assessment of the impact of active dendrites on LFPs account for synergistic interactions across all these pathological changes. Importantly, such analyses should account for the cell-type and location-dependent changes observed in ion channels, receptors, and connectivity from other cell types.

## Computational Methods for LFP Modeling

The mechanisms underlying the origins of LFPs are complex and remain open to exploration (Buzsaki et al., 2012; Einevoll et al., 2013). They involve specific spatiotemporal structures of synaptic inputs, which could be either from long-range afferents or from local synapses. These inputs lead to intra-/inter-cellular spatiotemporal responses from and interactions among intrinsic properties of neuronal dendrites and soma. These in turn generate transmembrane currents that ultimately shape the LFP. Furthermore, these synaptic inputs and intrinsic properties vary across different brain regions depending on varying cell morphologies and topographical arrangements (Buzsaki et al., 2012; Einevoll et al., 2013). In addition to such interregional differences, there are significant heterogeneities in neural properties in any given brain region, which can also undergo changes through neuromodulation, plasticity, or pathological conditions. The fundamental question on the specific contributions of the plethora of active dendritic conductances to LFPs, and how they change in the face of all the gradients, heterogeneities, and dynamics (highlighted in the previous sections) continues to remain largely unexplored. In the face of such complexity, biophysically rooted theoretical and computational modeling tools have become invaluable for uncovering the mechanisms underlying the generation of various forms of LFP – fast *vs*. slow, rhythmic *vs*. irregular, unitary *vs*. population, synchronous *vs*. asynchronous across multiple locations, highly localized vs. travelling, etc. These tools are also useful for explaining new findings, and in delineating the relative contributions of different cellular and network elements to field potentials (Rall, 1962; Einevoll et al., 2013; Pesaran et al., 2018; Einevoll et al., 2019; Poirazi and Papoutsi, 2020). To highlight these tools, in the following subsections we review the 3 most commonly used approximation methods that are used in computational studies for modeling LFPs. We also point to various free and open-source resources that are available for those researchers who are interested in dabbling with LFP modeling.

## Point source approximation

**Fig. 4:**
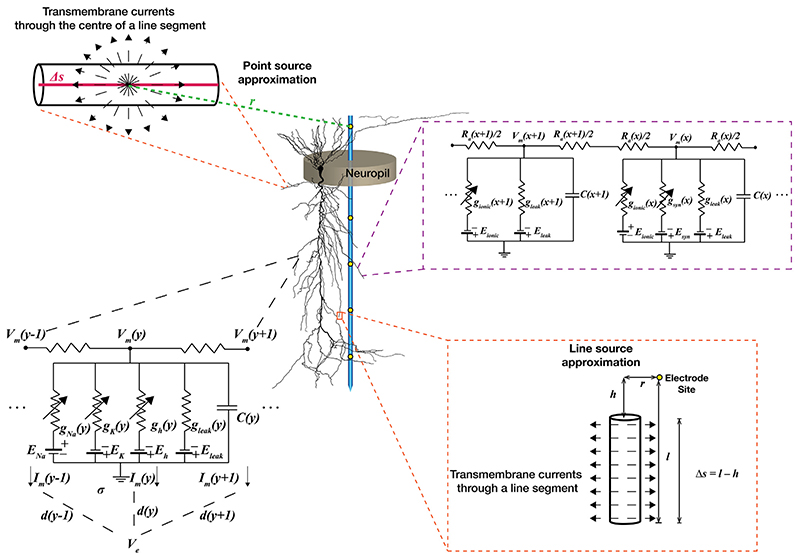
Approaches to account for active dendrites in LFP computation *Center*: A single electrode with multiple recording sites in the center of a cylindrical neuropil of a population of morphologically realistic pyramidal neurons. Note, while a single morphology is depicted here for simplicity, a heterogeneous set of morphologies should be employed to account for the underlying structural heterogeneity. *Top Left*: Point source approximation for modeling LFP. *Bottom Right*: Line source approximation for modeling LFP. *Top Right*: A representation of conductance-based mutli-compartmental model depicting how to account for location-dependent expression of passive leak, active (voltage-gated) ionic, and synaptic conductances. *Bottom Left*: A representation of a single compartment within the morphology and the transmembrane currents generated through it due to the presence of active and passive conductances. These transmembrane currents are used for computing LFPs. Symbols used: *r*: radial distance; *σ*^:^ macroscopic extracellular conductivity; *l*,*h*: longitudinal distance from the beginning and end of the line respectively; Δ*s*: length of line segment; *V_m_*: intracellular membrane potential; *I_m_*: transmembrane current; *x*/*y*: location variables; *R_a_*: axial resistance; *g*: conductance (subscript represents the type of conductance); *E*: reversal potential (subscript represents the type of conductance); *C*: capacitance; *d*: distance; *V_e_*: extracellular potential.

In this approximation method, the extracellular potential generated from a neuronal compartment is approximated to originate from a point source (Holt, 1998; Holt and Koch, 1999; Gold et al., 2006). The transmembrane currents are assumed to originate from a point in the center of a neuronal compartment (Fig. 4). Transmembrane currents are recorded from such point sources for each compartment across the three-dimensional space. For *n* point sources (indexed by *i*) at a distance *r* from the recording location with macroscopic conductivity *σ* (i.e. the whole neuropil is modeled as a homogeneous conductor), the estimated field potential (Φ_*FP*_) is calculated from the transmembrane current (*I*) as:

$$\Phi_{FP}=\sum_{i=1}^{n}\frac{I_{i}}{4\pi \sigma r_{i}}$$

### Line source approximation

Line source approximation (LSA) is one of the most-commonly used methods for forward modeling of LFPs. In this approach, the extracellular potential generated from a neuronal compartment is approximated to originate from a continuous distribution of the transmembrane currents generated from a line that passes through the central axis of the compartment (Fig. 4). Except for distances less than 1 μm away from the recording location (Rosenfalck, 1969; Trayanova et al., 1990; Gold et al., 2006), LSA implementations are known to closely approximate the extracellular signal, and have been employed and validated for hippocampal pyramidal neurons (Gold et al., 2006). For *n* line sources (indexed by *i*) of length Δ*s*, the field potential (Φ_*FP*_) is computed as a linear sum of the individual field potentials due to each line source as:

$$\Phi_{FP}=\sum_{i=1}^{n}\frac{I_{i}}{4\pi \sigma \Delta s_{i}}\log\frac{\sqrt{h_{i}^{2}+r_{i}^{2}}-h_{i}}{\sqrt{l_{i}^{2}+r_{i}^{2}}-l_{i}}$$

where *h* is the longitudinal distance from the end of the line, *l* = Δ*s* + *h* is the distance from the beginning of the line.

There are some important considerations that need to be accounted for while modeling and analyzing LFPs (Buzsaki et al., 2012; Einevoll et al., 2013; Pesaran et al., 2018). First, several models assume homogeneous and isotropic extracellular conductivity, and therefore consider a constant value for *σ* (typically 0.3–0.4 S/m) in the equations for Φ_FP_ above. However, there are lines of evidence for inhomogeneities in extracellular conductivity (Lopez-Aguado et al., 2001; Goto et al., 2010), which can be accounted for in modeling and understanding LFPs (Gold et al., 2006; Pettersen et al., 2006; Einevoll et al., 2007). Second, the use of *σ* for the conversion from currents to field potentials implicitly assumes that the neuropil is bereft of frequency-dependent reactances. There have been several studies assessing frequency-dependence of the extracellular space, both experimentally and computationally, with debates about the manifestion and the origins of frequency dependence (Bedard et al., 2004; Logothetis et al., 2007; Bedard et al., 2010; Gomes et al., 2016; Barbour, 2017; Bedard and Destexhe, 2017; Miceli et al., 2017; Pesaran et al., 2018). The broad consensus seems to be that the extracellular medium is largely resistive for the physiological range of signal frequencies (Pesaran et al., 2018). Finally, frequency-dependent features of LFPs should account for intrinsic dendritic membrane filtering and for correlation structures in synaptic inputs (Pettersen and Einevoll, 2008; Linden et al., 2011; Leski et al., 2013). Importantly, membrane filtering should not be assumed to be low-pass in nature (accounting only for the passive time constant), but should also account for location-dependent band-pass filters in neurons that manifest gradients in resonating conductances such as HCN channels (Narayanan and Johnston, 2007; Hu et al., 2009; Kalmbach et al., 2013; Sinha and Narayanan, 2015; Ness et al., 2016, 2018).

### Computing a proxy for LFP from point neuronal models

Several studies have used a linear summation of spiking dynamics of a neuronal network as a proxy for its LFP (Hagen et al., 2016; Telenczuk et al., 2020a; Martinez-Canada et al., 2021). A study showed that a specific linear combination of the synaptic currents from leaky integrate-and-fire (LIF) model neurons also provides a reasonable proxy for the LFP (Mazzoni et al., 2015). The spikes from LIF model neurons have also been used to stimulate bio-physically realistic neuron models in a hybrid scheme of LFP generation (Hagen et al., 2016; Skaar et al., 2020). It has also been suggested that the spikes of individual point neurons arranged in space can be convolved with their uLFPs and the linear summation of these uLFPs could be used to provide an imprecise but quick estimation of LFPs from an interconnected network of point neurons (Hagen et al., 2016; Telenczuk et al., 2020b). However, when it comes to understanding the mechanisms of LFP generation, such point neuron or single compartmental models may not fully account for the intricate spatiotemporal dynamics of synaptic inputs on the morphological details of dendrites. Having said that, there are neurons that are structurally and electrotonically compact, with the soma being the main site for synaptic inputs with very few dendritic arbors, such as those in the suprachiasmatic nucleus that is crucial for the maintenance of a 24 h circadian rhythm (Welsh et al., 2010; Harvey et al., 2020). For such neurons, the spiking activity of a network of point neurons could potentially be used as good estimate of the LFP as long as the point neurons are biophysically realistic and account for the diverse set of ion channels and receptors that these neurons express (Welsh et al., 2010; Harvey et al., 2020).

### Resources for modeling LFP

While the computational complexity for simulating LFPs is quite high, there are resources available to quickly get initiated. Extracellular Action Potential (EAP) simulations (Holt and Koch, 1999; Gold et al., 2006; Gold et al., 2007) written for NEURON® (Hines and Carnevale, 1997; Carnevale and Hines, 2006) and MATLAB® are available on ModelDB (Hines et al., 2004). While the original code was written to simulate EAPs, it can be easily adapted to model other extracellular phenomena. There are also several relatively new open-source libraries/software packages available to specifically model neuronal networks and LFPs: LFPsim (Parasuram et al., 2016), LFPy (Linden et al., 2014; Hagen et al., 2018), BioNet (Gratiy et al., 2018), NetPyNE (Dura-Bernal et al., 2019), Human Neocortical Neurosolver (HNN) (Neymotin et al., 2020) and Brain Modeling ToolKit (BMTK) (Dai et al., 2020).

## Coda and Future Directions

While spikes inarguably provide immense information about neurophysiology, not all events in the brain are above the spiking threshold of a neuron, especially in the dendrites and astrocytes. In addition, based on the ongoing activity and adaptation, the threshold itself can shift. Therefore, due credit must be given to dendritic subthreshold activity because many neuronal conductances, including ion channels, transporters, and receptors, are active in this subthreshold regime. They contribute to membrane potential dynamics and extracellular field potentials in both health and disease. Further, the brain is highly plastic, with heterogeneity and degeneracy emerging to be common phenomena across scales. Thus, the notion of assigning one-to-one functional mappings between mechanisms and their outcomes should be dealt with extreme caution and wherever applicable be replaced with that of many-to-one or even many-to-many mappings through rigorous computational and experimental analyses. Finally, computational studies should adopt a level of abstraction that is appropriate to answer the question at hand. If a model is being used to obtain a quick proxy for the LFP, point neuron network models with linear approximations may be used. But if the goal is to obtain mechanistic insights about the origins of LFPs, the model should incorporate both linear and non-linear biophysical details, not just at the soma but also in the dendrites, and across all cell types.

### Future directions for computational models: inverse meets forward to provide mechanistic insights

The first requirement for future computational models is to recognize the complexity underlying the emergence of LFPs, and not oversimplify LFP computations to be merely reflective of synaptic inputs. It is critical that insights about the complex computational capabilities of active dendritic structures, gained through electrophysiological experiments and computational models over the past few decades (Johnston et al., 1996; Segev and London, 2000; Migliore and Shepherd, 2002; Johnston et al., 2003; Johnston and Narayanan, 2008; Major et al., 2013; Grienberger et al., 2015; Stuart and Spruston, 2015; Payeur et al., 2019; Poirazi and Papoutsi, 2020), find their rightful place in defining network physiology and LFPs. Specifically, it is essential that theoretical frameworks and computational models incorporate the complexity of neurons endowed with active dendritic structures, explicitly accounting for the different sub- and supra-threshold conductances, their inter- and intra-neuronal gradients, different forms of modulation and plasticity, the ubiquitous heterogeneities spanning all neural components, the spatio-temporal interactions between different active dendritic components, and how they together drive network computations and LFPs. Given the expanding roles of glial cells in brain physiology (Araque et al., 2014; Ashhad and Narayanan, 2019; Santello et al., 2019; Kol and Goshen, 2020; Nagai et al., 2021), it is equally important to account for glial contributions to network computation and LFPs. To gain a holistic understanding of the origins and roles of LFPs, we recommend the amalgamation of inverse and forward models.

#### Inverse model

Using some preliminary knowledge about which groups of neurons are present in the circuit under investigation and their anatomical connectivity recurrent neural networks (RNNs), initially using random connectivity matrices, can be trained to match the experimentally obtained LFP data recorded from model animals and humans. Using the predictions from these RNNs mechanistic insights about the anatomical and functional connectivity between the neuronal circuit elements and their dynamics under different behavioral paradigms could be obtained (Rajan et al., 2016; Barak, 2017; Vu et al., 2018; Skaar et al., 2020). Further, where anatomical data is not available forward models can employ the connectivity insights obtained from these RNNs. We envision that such RNNs and other machine learning algorithms will prove to be useful for understanding the role and cellular/network origins of LFPs recorded from human healthy subjects and patients under different behavioral conditions, especially since molecular mechanistic insights may be difficult to obtain in these cases.

#### Forward model

There are several large-scale models of brain tissue and neuronal databases already available that can prove to be great resources for modeling LFP (Hines et al., 2004; Ascoli et al., 2007; Markram et al., 2015; Bezaire et al., 2016; Arkhipov et al., 2018; Gouwens et al., 2019; Billeh et al., 2020). Using morphologically and biophysically realistic models based on experimentally known intrinsic properties of the neurons and glial cells and their connectivity patterns, transmembrane currents could be computed under different input conditions to generate LFPs and gain insights about the collective roles of intrinsic/synaptic/morphological properties and spatio-temporal dynamics of inputs. Further virtually knocking-out individual intrinsic and synaptic mechanisms, introducing intrinsic/synaptic plasticity, synaptic scaling, and dendritic remodeling could help delineate their specific roles in the generation of LFPs under physiological and pathological conditions. From these insights, testable hypotheses can be obtained and experimentally verified using the extensive set of tools available for neuroscience research (Rivnay et al., 2017; Mendrela et al., 2018; Kim et al., 2020; Moreaux et al., 2020; Nectow and Nestler, 2020; Pal and Tian, 2020; Sabatini and Tian, 2020).

Finally, we strongly urge experimental and computational neuroscientists to collaborate extensively. Through such collaborations, elegant models may be obtained to understand the function of active dendrites and LFPs, and eventually their role in mediating behavior.

## Abbreviations

BMTK: brain modeling toolkit
dCaAP: dendritic calcium-mediated action potential
EAP: extracellular action potential
ECoG: electrocorticogram
EEG: electroencephalogram
EPSP: excitatory post-synaptic potential
ER: endoplasmic reticulum
GA: genetic algorithm
GABA: gamma-aminobutyric acid
GC: granule cell
HCN channels: hyperpolarization-activated cyclic nucleotide-gated cation non-specific channels
HNN: human neocortical neurosolver
InsP_3_R: inositol triphosphate receptors
LFP: local field potential
LSA: line source approximation
MPMOSS: multi-parametric multi-objective stochastic search
NMDA: N-methyl D-aspartate
NREM: non rapid eye movement
nRt: nucleus reticularis thalami
PFC: prefrontal cortex
REM: rapid eye movement
RNN: recurrent neural network
SEP: slow excitatory potential
SERCA: sarco/endoplasmic reticulum Ca^2+^- ATPase
SPW-R: sharp wave ripple complex
STA-LFP: spike-triggered averaged local field potential
TC: thalamocortical
uLFP: unitary local field potential.
